# Fairness of artificial intelligence in healthcare: review and recommendations

**DOI:** 10.1007/s11604-023-01474-3

**Published:** 2023-08-04

**Authors:** Daiju Ueda, Taichi Kakinuma, Shohei Fujita, Koji Kamagata, Yasutaka Fushimi, Rintaro Ito, Yusuke Matsui, Taiki Nozaki, Takeshi Nakaura, Noriyuki Fujima, Fuminari Tatsugami, Masahiro Yanagawa, Kenji Hirata, Akira Yamada, Takahiro Tsuboyama, Mariko Kawamura, Tomoyuki Fujioka, Shinji Naganawa

**Affiliations:** 1 Department of Diagnostic and Interventional Radiology, Graduate School of Medicine, Osaka Metropolitan University, 1-4-3 Asahi-Machi, Abeno-ku, Osaka, 545-8585 Japan; 2 STORIA Law Office, Chuo-ku, Kobe, Hyogo Japan; 3 Department of Radiology, University of Tokyo, Bunkyo-ku, Tokyo, Japan; 4 Department of Radiology, Juntendo University Graduate School of Medicine, Bunkyo-ku, Tokyo, Japan; 5 Department of Diagnostic Imaging and Nuclear Medicine, Kyoto University Graduate School of Medicine, Sakyoku, Kyoto Japan; 6 Department of Radiology, Nagoya University Graduate School of Medicine, Nagoya, Aichi Japan; 7 Department of Radiology, Faculty of Medicine, Dentistry and Pharmaceutical Sciences, Okayama University, Kita-ku, Okayama, Japan; 8 Department of Radiology, Keio University School of Medicine, Shinjuku-ku, Tokyo, Japan; 9 Department of Diagnostic Radiology, Kumamoto University Graduate School of Medicine, Chuo-ku, Kumamoto, Japan; 10 Department of Diagnostic and Interventional Radiology, Hokkaido University Hospital, Sapporo, Japan; 11 Department of Diagnostic Radiology, Hiroshima University, Minami-ku, Hiroshima, Japan; 12 Department of Radiology, Osaka University Graduate School of Medicine, Suita City, Osaka Japan; 13 Department of Diagnostic Imaging, Graduate School of Medicine, Hokkaido University, Kita-ku, Sapporo, Hokkaido Japan; 14 Department of Radiology, Shinshu University School of Medicine, Matsumoto, Nagano Japan; 15 Department of Diagnostic Radiology, Tokyo Medical and Dental University, Bunkyo-ku, Tokyo, Japan

**Keywords:** Fairness, Bias, Artificial intelligence, Healthcare, Medicine, Review

## Abstract

In this review, we address the issue of fairness in the clinical integration of artificial intelligence (AI) in the medical field. As the clinical adoption of deep learning algorithms, a subfield of AI, progresses, concerns have arisen regarding the impact of AI biases and discrimination on patient health. This review aims to provide a comprehensive overview of concerns associated with AI fairness; discuss strategies to mitigate AI biases; and emphasize the need for cooperation among physicians, AI researchers, AI developers, policymakers, and patients to ensure equitable AI integration. First, we define and introduce the concept of fairness in AI applications in healthcare and radiology, emphasizing the benefits and challenges of incorporating AI into clinical practice. Next, we delve into concerns regarding fairness in healthcare, addressing the various causes of biases in AI and potential concerns such as misdiagnosis, unequal access to treatment, and ethical considerations. We then outline strategies for addressing fairness, such as the importance of diverse and representative data and algorithm audits. Additionally, we discuss ethical and legal considerations such as data privacy, responsibility, accountability, transparency, and explainability in AI. Finally, we present the Fairness of Artificial Intelligence Recommendations in healthcare (FAIR) statement to offer best practices. Through these efforts, we aim to provide a foundation for discussing the responsible and equitable implementation and deployment of AI in healthcare.

## Introduction

Fairness is one of the core principles of artificial intelligence (AI) ethics [1–3], and in recent years, there has been an increase in efforts focusing on fairness in AI, with a growing number of publications highlighting the need for improvement [4–9]. Various biases are involved in developing and applying AI, and these biases can affect fairness by erroneously skewing AI results [10]. In the medical field, bias and discrimination in AI have been studied in various domains [11, 12]. The World Medical Association's Geneva Declaration cites factors such as “age, disease or disability, creed, ethnic origin, gender, nationality, political affiliation, race, sexual orientation, social standing or any other factor” as examples that should not influence a physician’s obligation to their patients [13]. Therefore, fairness concerns arise if AI does not perform adequately for specific patients.

AI research in radiology is an active field in healthcare owing to its affinity for imaging [14], with the number of AI-related publications and medical device certifications increasing annually [15–17]. One important reason for this is the global shortage of radiologists [18–21]. In particular, Japan has numerous publications on AI use in the field of radiology, including X-ray [22–25], mammography [26–30], US [31], CT [32–40], MRI [41–50], and PET [51, 52]. This surge in Radiological AI publications in Japan could be related to Japan having both the lowest number of radiologists per capita and the highest number of CT and MRI machines per capita among the Organization for Economic Co-operation and Development (OECD) countries [53]. Furthermore, owing to the coronavirus disease 2019 (COVID-19) pandemic, the number of COVID-19-related studies from Japan has increased [54–61], with a significant increase in such research focusing on AI [62–64].

As physicians in this era of AI clinical practice, we must be mindful of fairness concerns arising from AI bias in healthcare to provide better care to all patients. This review aims to provide a comprehensive overview of concerns related to AI fairness, discuss strategies to mitigate AI biases, and emphasize the need for collaboration among stakeholders to ensure equitable AI integration. In doing so, it lays the foundation for discussing the responsible and equitable implementation and deployment of AI in healthcare.

First, fairness in healthcare is discussed. We then discuss the issue of bias in AI systems used in healthcare. Next, we suggest strategies to reduce bias such as using diverse data, validating algorithms, and educating clinicians and patients regarding AI. We then discuss ethical and legal issues such as patient consent, data privacy, accountability, and the need for transparency in AI systems. Collaboration is key in this context; therefore, we explore the roles of various stakeholders, including physicians, AI researchers, policymakers, regulatory authorities, patients, advocacy groups, and professional associations. We include the best practices recommended for fairness in AI and the areas where more research is needed. Finally, we conclude the paper with a summary of our main findings.

## Fairness concerns in healthcare

### Defining fairness in healthcare

Fairness in healthcare is a multidimensional concept that includes the equitable distribution of resources, opportunities, and outcomes among diverse patient populations [65]. The concept of fairness is based on the fundamental ethical principles of justice, beneficence, and non-maleficence. Healthcare systems must provide access to high-quality care for all individuals without discrimination. In the context of radiology, fairness in AI refers to the development and deployment of unbiased AI that provides accurate diagnoses and treatments for all patients regardless of their social status or ethnic differences. Achieving this fairness requires a comprehensive understanding of the potential causes of bias in AI and development of strategies to mitigate these biases [66].

### Biases of AI in healthcare

**Fig. 1:**
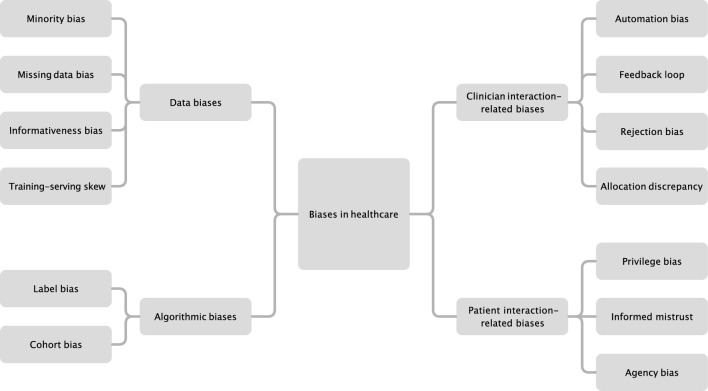
Biases in healthcare of AI

Generally, biases in AI can emerge from two main sources: the data used for algorithm training (data bias) and inherent design or learning mechanisms of the algorithm itself (algorithmic bias). However, in the healthcare context, additional biases may arise because of the complex nature of human interactions and decision-making processes. These additional biases can be classified into two types: those that originate from AI–clinician interactions and those that originate from AI–patient interactions [11]. An overview of these biases is shown in Fig. 1.

### Data biases

Data bias refers to problems arising from the collection and organization of data used in AI training that can potentially have harmful effects on fairness and accuracy [67]. The types of data biases include minority bias, missing data bias, informativeness bias, and training–serving skew [11]. Minority bias occurs when the number of protected group members in the dataset is insufficient for AI to learn accurate statistical patterns. This can lead to decreased performance and biased results when the algorithm is applied to these underrepresented groups. For example, many cardiovascular risk prediction algorithms have a history of being trained primarily on male patient data [68, 69]. This has led to an inaccurate risk assessment in female patients with different symptoms and risk factors. Missing data bias occurs when data from protected groups are missing nonrandomly, making it difficult for AI to generate accurate predictions. For example, if patients in contact isolation have fewer vital sign records than other patients, the algorithm may struggle to identify clinical deterioration. Informativeness bias occurs when the features used for detection are not as apparent for certain protected groups, lowering their informativeness when predictions are made. For example, identifying melanoma from images of patients with dark skin is more challenging than those with light skin [70, 71]. Training–serving skew refers to the mismatch between the data used for AI training and those used during deployment. This can arise from non-representative training data due to selection bias or from the deployment of the model on patients with a population prevalence different from that of the training data. In a study training AI to diagnose pneumonia from chest X-rays, the performance on unseen data from the institution where the training data were collected was significantly higher than its performance on data collected from external hospitals [72]. This common scenario means that estimations of AI performance based on internal test data may overestimate its real-world performance on external data [73–75].

### Algorithmic biases

Algorithmic bias refers to problems arising from the development and implementation of AI, which can negatively affect fairness and effectiveness. Even with representative data without data bias, AI can exhibit bias because of its inherent design or learning mechanisms. Algorithmic biases include label and cohort bias [11]. Label bias is a broad concept that includes test referral and interpretation bias. This occurs when AI training uses inconsistent labels, which may be influenced by healthcare disparities rather than universally accepted truths. This can lead to biased decision-making based on inaccurate or inconsistent information in the AI algorithms. For example, significant racial bias has been observed in commercially available algorithms used to predict patients' healthcare needs. Although several biases were affected, a major contributing factor to this algorithm’s bias was its design, which used cost as a proxy for healthcare needs, leading to an underestimation of the needs of Black patients compared with those who are White with similar conditions [76]. Cohort bias occurs when AI is developed based on traditional or easily measurable groups without considering other potentially protected groups or varying levels of granularity. For example, mental health disorders have been underdiagnosed or misdiagnosed within lesbian, gay, bisexual, transgender, queer or questioning, intersex, asexual, and other (LGBTQ +) populations [77]. One reason for this is that algorithms often do not take the granularity of the LGBTQ + population into account and rely only on information about biological males and females. AI trained on such data may continue to overlook or misdiagnose mental health issues in these populations, potentially perpetuating existing disparities in mental healthcare.

### Clinician interaction-related biases

When healthcare professionals interact with AI, biases related to interactions can occur, potentially affecting the algorithm's performance, fairness, and adoption [11]. One such bias is automation bias, which refers to the tendency to overly rely on AI when tasks are transferred from healthcare professionals to AI programs [78]. Overconfidence in algorithms can result in inappropriate actions based on inaccurate predictions. One study found that incorrect AI advice negatively affected radiologists' mammogram reading performance across all expertise levels. Inexperienced radiologists are more likely to follow incorrect AI suggestions [79]. Another bias related to interactions is the feedback loop [80]. This occurs when clinicians accept AI recommendations even if they are incorrect, leading the algorithm to relearn and perpetuate the same mistakes. Rejection bias refers to the conscious or unconscious desensitization to excessive alerts. Alert fatigue is a manifestation of this bias, as clinicians may ignore important alerts owing to an overwhelming number of false alarms [81, 82]. Finally, an allocation discrepancy occurs when the positive predictive values for protected groups are disproportionately low, leading the AI to withhold necessary resources, such as clinical attention or social services. Such resource allocation discrepancies can exacerbate disparities in care and outcomes among the affected groups.

### Patient interaction-related biases

Biases related to interactions between patients and AI or the systems that incorporate them include privilege bias, informed mistrust, and agency bias [11]. Privilege bias occurs when certain populations cannot access AI in care settings or when these algorithms require technology or sensors that are not available to all populations [83]. This can lead to an unequal distribution of AI-driven healthcare benefits, potentially exacerbating existing healthcare disparities. Informed mistrust refers to the skepticism protected groups may have toward AI owing to historical exploitation and unethical practices in healthcare [84, 85]. This mistrust may lead these patients to avoid care or intentionally conceal information from clinicians or systems using AI. Agency bias arises when protected groups lack a voice in the development, use, and evaluation of AI [86]. These groups may lack the access, resources, education, or political influence necessary to detect AI biases, voice concerns, and affect change. This lack of agency can result in AI inadequate at considering the needs and perspectives of protected groups, potentially leading to biases and disparities in healthcare outcomes.

### Strategies to mitigate bias

#### Diverse and representative data

One of the most effective methods of mitigating AI biases is to ensure the use of diverse and representative datasets during AI development and training [67, 87]. This process entails carefully collecting and incorporating data from a wide range of sources to accurately reflect the demographics, characteristics, healthcare needs, and potential disparities in the target population. This diversity is not only critical for developing AI systems capable of catering to a multitude of patient requirements but also for fostering trust and confidence in AI-driven healthcare solutions. By incorporating data from various patient populations, age groups, disease stages, cultural and socioeconomic backgrounds, and healthcare settings, AI can learn to recognize, diagnose, and treat a broad spectrum of patient conditions with greater precision and contextual understanding. This comprehensive approach to data collection and curation prevents potential biases from occurring in AI systems, resulting in a reduction of disparities and promotion of equity in healthcare outcomes [76]. Furthermore, a diverse and representative dataset ensures that AI algorithms are rigorously tested across different scenarios, thereby enhancing their overall performance and utility. This enables healthcare providers to rely on AI-driven diagnostics and treatment recommendations, leading to improved patient care and reduced clinician workload.

#### Algorithm auditing and validation

Regular audits and AI validation play crucial roles in identifying and addressing potential biases and ensuring that AI systems remain fair, accurate, and effective in diverse healthcare settings. Independent audits by external experts or organizations can be conducted to evaluate the fairness, accuracy, and performance of AI, with adjustments made to the algorithms to correct identified biases [88]. The healthcare landscape is constantly changing; therefore, there is no guarantee that an AI algorithm with high performance will maintain its high performance in the future [89]. Validation studies are essential for verifying the effectiveness of AI in different patient populations and conditions [72]. The establishment of a dedicated department within hospitals for algorithm quality control has been advocated [90]. This department should be responsible for continuously monitoring AI performance, identifying potential biases, and making the necessary updates to algorithms. This proactive approach to quality control would ensure that AI systems are held accountable and maintain their effectiveness in providing accurate and equitable care for all patients. Considering the growing prevalence of medical AI, practitioners must remain vigilant and evaluate key indicators, such as underdiagnosis rates and other health disparities, during the algorithm development process and after deployment. This ongoing evaluation will help identify and rectify emerging issues, ensuring that AI systems continue to serve patients effectively and equitably.

#### Education to both clinicians and patients

Educating clinicians and patients on the biases inherent in AI is crucial for fostering a shared understanding and promoting fairness in healthcare [1]. This educational process involves raising awareness of potential biases, sharing best practices to address them, and encouraging open discussions on the implications of AI in healthcare decision-making. Clinicians aware of AI biases can avoid overreliance on AI-generated results and make decisions based on more accurate information [91]. This increased awareness enables healthcare professionals to critically evaluate AI recommendations, weigh potential risks and benefits, and consider alternative sources of information when making patient care decisions. Additionally, clinicians can advocate for, and participate in, the development and evaluation of AI systems to ensure that their expertise and experience are incorporated into the models, further enhancing their accuracy and reliability. Patients who understand AI biases can make more informed and satisfactory decisions [92, 93]. By being aware of the potential limitations and biases of AI-generated recommendations, patients can engage in more meaningful conversations with their healthcare providers regarding treatment options and play a more active role in their care. This empowerment promotes patient-centered care and ensures that individual preferences, values, and circumstances are considered when making healthcare decisions. To foster a culture of continuous learning and improvement of AI, creating channels for feedback and collaboration among healthcare professionals and patients is essential. This can be achieved through workshops, conferences, online forums, or interdisciplinary collaborations that bring together diverse perspectives and experiences. By sharing knowledge, insights, and best practices, they can work together to identify and address biases and continuously refine AI systems to better serve the needs of all patients.

### Ethical and legal considerations

#### Data privacy and security

Ensuring data privacy is an important ethical and legal consideration for AI fairness, as it has a significant impact on patient autonomy, trust in AI, and compliance with legal frameworks. Respecting patient autonomy and protecting confidential medical information is the foundation of ethical AI implementation, which can only be achieved by addressing important issues related to data privacy [94, 95]. One such issue is obtaining informed consent for data use [96, 97]. Patients must fully understand how their data are used, shared, and stored by AI. To achieve this, transparent communication regarding the purpose, risks, and benefits of data sharing is required to enable patients to make informed decisions regarding participating in AI-driven healthcare initiatives. Protecting data storage and transmission is an important aspect of data privacy [98]. Robust security measures, such as encryption and anonymization techniques, are required to protect patient data from unauthorized access, data breaches, and other cybersecurity threats. Moreover, strict access controls and audit mechanisms must be implemented to monitor and track data use, ensure accountability, and prevent data misuse. Compliance with privacy regulations, such as the Health Insurance Portability and Accountability Act (HIPAA) in the United States and the General Data Protection Regulation (GDPR) in the European Union, is essential for legally and ethically sound AI practice [99]. These regulations provide strict guidelines for the collection, storage, and processing of personal health information, and AI researchers and healthcare professionals must adhere to standardized data protection protocols. By addressing these challenges and ensuring data privacy, AI developers and healthcare professionals can foster trust in AI, maintain patient autonomy, and adhere to ethical and legal standards. This promotes the development and implementation of fair and equitable AI-driven healthcare solutions that respect the privacy and dignity of all patients.

#### Liability and accountability

To address the potential errors, harmful outcomes, and biases in the predictions generated by AI, clear guidelines for responsibility and accountability in healthcare AI must be established. This includes determining the roles and responsibilities of various stakeholders, such as physicians, AI developers, and healthcare institutions, in cases where misdiagnoses or other patient harm occur [100, 101]. Physicians should be responsible for verifying AI-generated diagnoses and integrating them into the clinical decision-making process. This may involve critically evaluating the AI outputs, considering them along with other relevant clinical information, and making informed decisions regarding patient care. Conversely, AI developers have a responsibility to ensure the accuracy, reliability, and fairness of their algorithms. This includes addressing biases and continuously improving the algorithms based on feedback from the clinical community. Developers also need to provide clear guidance on the intended use and limitations of AI solutions, enabling physicians to make informed judgments regarding their application in patient care. Healthcare institutions play a crucial role in overseeing the integration of AI solutions into clinical workflows [90]. They must ensure that the necessary infrastructure, training, and support are available for the safe and effective use of AI. This includes developing policies and procedures for managing potential risks and harmful outcomes as well as monitoring and evaluating AI performance to ensure continuous quality improvement. A robust framework for accountability and responsibility enables AI stakeholders to address potential ethical and legal issues more effectively. As a result, trust in AI-driven healthcare solutions can increase, fostering responsible use and improving patient outcomes and overall care quality.

#### Transparency and explainability

Transparency and accountability are essential elements of ethical AI as they enable healthcare professionals and patients to understand the basis of AI-generated predictions and foster trust in AI [102]. To enhance the accountability of AI, developing interpretable algorithms, visualizing decision-making processes, and providing comprehensible explanations for AI predictions are important [103]. By improving transparency and accountability in AI in healthcare, both healthcare professionals and patients can be supported in making informed decisions, and ethical and legal concerns associated with the use of AI in healthcare can be addressed [104]. However, recognizing the limitations of explainability is important [104–107]. Even with saliency maps that visualize the areas of an image contributing to AI judgment, humans must decipher the meaning behind the explanation. When people favor meanings that confirm their beliefs or hypotheses, this is called confirmation bias. In other words, humans tend to interpret explanations positively even if the AI is not accurate or trustworthy. Recognizing the limitations of explainable AI is important for maintaining a realistic perspective of its potential benefits and drawbacks. By striking a balance among transparency, accountability, and understanding the limitations of explainable AI, healthcare professionals can address the ethical and legal concerns associated with the use of AI in healthcare.

### Collaboration among stakeholders

#### Physicians, AI researchers, and AI developers

Collaboration among physicians, AI researchers, and AI developers is essential to address fairness concerns in AI [108, 110]. Physician participation can provide valuable domain expertise and insights for AI researchers. Recent AI, developed to utilize images [14, 111], and radiologists are particularly well matched. A cycle of improvement can be achieved through communicating expertise in the field with physicians and sharing their experience in using AI in actual medical practice. By working together, stakeholders can identify potential biases and develop effective strategies to mitigate them, ensuring that AI is fair, equitable, and effective. Additionally, empirical research on AI biases is often difficult for independent researchers to analyze as large-scale deployed algorithms are generally proprietary [112]. This makes it difficult to adequately assess bias, and active collaboration of not only AI researchers but also AI developers from companies is essential.

#### Policymakers and regulatory authorities

Policymakers and regulatory authorities play a crucial role in ensuring AI fairness through establishing comprehensive guidelines, standards, and regulations that govern the development and deployment of AI in healthcare [95, 113–115]. Through proactively shaping policies, they can promote the development of frameworks for AI design, training, and validation, ensuring that AI is built with fairness and inclusivity in mind. Fostering transparency and accountability in AI is also an important aspect of their responsibilities [5, 116]. Policymakers and regulatory authorities can implement requirements for AI developers to disclose their methodologies, data sources, and performance metrics, allowing for a better evaluation and comparison of AI. Furthermore, policymakers and regulatory authorities can allocate resources and funding towards AI innovation and research as well as towards addressing issues on fairness and equity in AI-driven healthcare. Through formulating policies that encourage the development of AI technologies focused on health equity, policymakers and regulators can minimize bias and ensure that all patients benefit from AI, regardless of their background or circumstances. This will contribute to a more equitable healthcare system, in which AI-driven solutions can improve patient outcomes and reduce disparities in access to high-quality care.

#### Patients and advocacy groups

Patients and advocacy groups serve a crucial function in advancing AI fairness as they contribute valuable insights and firsthand experiences to the conversation. They can provide valuable insights into the needs and preferences of diverse patient populations, ensuring that AI addresses the specific challenges faced by various communities [86, 117, 118]. As patients directly affected by the AI output, they have a vested interest in identifying areas in which AI may be subject to potential biases and disparities in healthcare outcomes. By collaborating with patients and advocacy groups, physicians, AI researchers, and AI developers can gain a deeper understanding of the unique challenges and concerns faced by various patient populations and promote the development of more equitable and effective AI solutions tailored to individual needs [119]. This can also help build trust in AI-driven healthcare [84, 85]. By giving them a voice in the design, implementation, and evaluation of AI, organizations can demonstrate their commitment to patients by addressing their concerns and enhancing transparency.

#### Professional associations

Professional associations are pivotal in steering the development and implementation of AI-driven healthcare solutions, addressing ethical challenges, and promoting best practices. Establishing guidelines, standards, and ethical frameworks, fostering interdisciplinary collaborations, and facilitating open dialogue among all stakeholders will bridge this gap. Their unique position allows them to contribute to the development of fair and transparent policies and practices while ensuring that AI technologies are developed and deployed responsibly, equitably, and in the best interests of patients.

### Recommendations and future directions

#### Best practices in healthcare for fairness of AI

To promote AI equity in healthcare and ensure fair and accurate care for all patients, developing a comprehensive strategy that addresses biases at multiple levels as well as ethical, legal, and practical concerns is essential. This approach should foster collaboration among key stakeholders to achieve equitable AI-driven healthcare solutions. We present the following recommendations, called the FAIR (Fairness of Artificial Intelligence Recommendations in healthcare) principles, which aim to ensure fair and equitable AI-driven healthcare solutions (see Table 1):

1. Ensuring diverse and representative data in AI development

**Table 1:** FAIR (Fairness of Artificial Intelligence Recommendations in healthcare) statement

1. Ensuring diverse and representative data in AI development
Utilize diverse and representative data during AI development and training. This will ensure that AI systems can better recognize, diagnose, and treat a wide range of patient conditions, reducing disparities and promoting equity in healthcare outcomes
2. Independent audits and validation of AI algorithms
Implement regular audits and validation of AI algorithms by independent experts or organizations. This will ensure objectivity and transparency in the evaluation process and help identify potential biases, leading to necessary adjustments in the algorithms. Establish a dedicated system within hospitals for algorithm quality control to continuously monitor AI performance, identify potential biases, and update algorithms accordingly
3. Education on AI biases for clinicians and patients
Educate clinicians and patients on the biases inherent in AI with ongoing education as needed. This will promote a shared understanding and encourage open discussions on the implications of AI in healthcare decision-making by creating channels for feedback and collaboration among healthcare professionals and patients. This can be achieved through workshops, conferences, online forums, and interdisciplinary collaborations
4. Strengthening data privacy and security measures
Strengthen data privacy and security measures, ensuring compliance with existing legal frameworks such as HIPAA and GDPR. Develop transparent communication protocols to educate patients regarding data usage, storage, and sharing, allowing them to make informed decisions regarding participating in AI-driven healthcare initiatives
5. Establishing liability and accountability frameworks
Establish a robust framework for liability and accountability, clearly defining the roles and responsibilities of physicians, AI developers, and healthcare institutions. Encourage continuous feedback and improvement of AI algorithms, while maintaining transparency and providing guidance on AI solutions' intended use and limitations
6. Enhancing AI transparency and explainability
Enhance transparency and explainability in AI by developing interpretable algorithms, visualizing decision-making processes, and providing understandable explanations for AI predictions. Recognize the limitations of explainable AI and address potential biases to prevent overreliance on AI-generated outputs
7. Collaboration between physicians, AI researchers, and developers
Foster collaboration between physicians, AI researchers, and developers to share expertise, identify potential biases, and develop strategies to mitigate them. Encourage active participation of AI companies to support independent research on AI biases and improve algorithm fairness
8. Policymaker and regulatory authority involvement
Engage policymakers and regulatory authorities in developing comprehensive guidelines, standards, and regulations to ensure AI fairness, promote transparency and accountability, and allocate resources to support research and innovation in AI-driven healthcare
9. Patient and advocacy group participation in AI development and evaluation
Involve patients and advocacy groups in the design, implementation, and evaluation of AI solutions, giving them a voice in the decision-making process. Leverage their insights and experiences to address unique challenges and promote the development of equitable AI solutions tailored to individual needs
10. Professional association support
Professional associations help establish guidelines, standards, and ethical frameworks, and promote interdisciplinary collaborations and open discussions among all stakeholders. Their unique position enables them to aid in creating fair and transparent policies and practices

*AI* artificial intelligence, *HIPAA* the health insurance portability and accountability act, *GDPR* the general data protection regulation

Utilize diverse and representative data during AI development and training. This ensures that AI systems can better recognize, diagnose, and treat a wide range of patient conditions, reduce disparities, and promote equity in healthcare outcomes.

2. Independent audits and validation of AI algorithms

Implement regular audits and validate AI algorithms by independent experts or organizations. This ensures objectivity and transparency in the evaluation process and helps identify potential biases, leading to necessary adjustments in the algorithms. Establish a dedicated system within hospitals for algorithm quality control to monitor AI performance continuously, identify potential biases, and update algorithms accordingly.

3. Education on AI biases for clinicians and patients

Educate clinicians and patients on the biases inherent in AI with ongoing education as needed. This will promote a shared understanding and encourage open discussions on the implications of AI in healthcare decision-making by creating channels for feedback and collaboration among healthcare professionals and patients. This can be achieved through workshops, conferences, online forums, and interdisciplinary collaborations.

4. Strengthening data privacy and security measures

Strengthen data privacy and security measures, ensuring compliance with existing legal frameworks such as HIPAA and GDPR. Develop transparent communication protocols to educate patients regarding data usage, storage, and sharing, allowing them to make informed decisions regarding participating in AI-driven healthcare initiatives.

5. Establishing liability and accountability frameworks

A robust framework for liability and accountability should be established, clearly defining the roles and responsibilities of physicians, AI developers, and healthcare institutions. Encourage continuous feedback and improvement of AI algorithms while maintaining transparency and providing guidance on the intended use and limitations of AI solutions.

6. Enhancing AI transparency and explainability

Enhance transparency and explainability in AI by developing interpretable algorithms, visualizing the model’s decision-making processes, and providing explanations for AI predictions. Recognize the limitations of explainable AI and address potential biases to prevent overreliance on AI-generated outputs.

7. Collaboration between physicians, AI researchers, and developers

Foster collaboration among physicians, AI researchers, and developers to share expertise, identify potential biases, and develop strategies to mitigate them. Active participation of AI companies should be encouraged to support independent research on AI biases and improve algorithm fairness.

8. Policymaker and regulatory authority involvement

Engage policymakers and regulatory authorities in developing comprehensive guidelines, standards, and regulations to ensure AI fairness; promote transparency and accountability; and allocate resources to support research and innovation in AI-driven healthcare.

9. Patient and advocacy group participation in AI development and evaluation

Involve patients and advocacy groups in the design, implementation, and evaluation of AI solutions, giving them a voice in the decision-making process. Leverage their insights and experiences to address unique challenges and promote the development of equitable AI solutions tailored to individual needs.

10. Professional association support
  Professional associations help establish guidelines, standards, and ethical frameworks, and promote interdisciplinary collaborations and open discussions among all stakeholders. Their unique position enables them to create fair and transparent policies and practices.

By﻿ implementing these recommendations and addressing biases in data and algorithms, stakeholders in the AI-driven healthcare sector can foster trust, transparency, and inclusivity. This will ensure that AI technologies are developed and deployed ethically, responsibly, and equitably for the benefit of all patients regardless of their differences. Ultimately, this approach contributes to a more equitable healthcare system and improves patient outcomes.

#### Research gaps and future work

Several research gaps and opportunities for future research to address concerns regarding AI bias and fairness exist. Randomized controlled trials should be conducted to explore the potential of AI in improving patient care and outcomes. These trials should include diverse populations and AI that are tailored to the specific needs of different demographic groups. The long-term impact of AI adoption in healthcare on patient treatment, outcomes, and physician workload should be investigated. The models should be monitored regularly to address biases that may emerge over time [89]. Developing new technologies for explainability and transparency is necessary to enable healthcare professionals and patients to better understand AI-generated predictions, foster trust in AI, and ensure its ethical deployment.

## Conclusion

In this review, we first defined fairness in AI in the healthcare domain, introduced various biases with examples and potential countermeasures, and emphasized the importance of collaborating with stakeholders. Subsequently, we discussed important ethical and legal issues. As a result, we summarized the best practices into the FAIR statement. This includes preparing diverse and representative data, continuously validating AI, educating physicians and patients, and emphasizing the importance of interdisciplinary collaboration. Although implementing each best practice is difficult, these efforts have become increasingly important as AI integration advances in the medical field. Furthermore, AI technology is still evolving, and the situation is constantly changing, with new challenges emerging one after another. The emergence of tools like Chat Generative Pre-trained Transformer (ChatGPT) is expected to greatly change white-collar jobs, and physicians are no exception [120–122]. We are currently in an era in which physicians require flexible thinking and the ability to respond quickly to new technologies.

Since its inception, AI has influenced several aspects of modern society, leading to notable advancements. The medical field has not remained untouched by this wave of change, with radiology particularly poised to harness the power of AI [123]. Given this unique position, the radiology community has a vital responsibility to share its experience in actively integrating AI into medicine [124–126], providing invaluable guidance and insight for other medical specialties. As pioneers in the implementation of AI, radiologists should champion AI equity in healthcare. Our early experience navigating the complex landscape of AI adoption and overcoming the challenges associated with its deployment can serve as a roadmap for other medical professionals. By doing so, we can ensure that AI benefits all patients, regardless of their backgrounds, and contributes to the greater good of society.
